# Comparison of the effects of gelatin, Ringer’s solution and a modern hydroxyl ethyl starch solution after coronary artery bypass graft surgery

**DOI:** 10.5830/CVJA-2012-026

**Published:** 2012-09

**Authors:** SM Alavi, T Babaei, B Baharvand Ahmadi, B Baharestani

**Affiliations:** Department of Anaesthesiology and Intensive Care Medicine, Rajaei Heart Centre, Tehran University of Medical Sciences, Tehran, Iran; Department of Anaesthesiology and Intensive Care Medicine, Rajaei Heart Centre, Tehran University of Medical Sciences, Tehran, Iran; Department of Cardiovascular Surgery, Rajaei Heart Centre, Tehran University of Medical Sciences, Tehran, Iran; Department of Cardiovascular Surgery, Rajaei Heart Centre, Tehran University of Medical Sciences, Tehran, Iran

**Keywords:** CABG, haemodynamic stability

## Abstract

**Objective:**

The aim of this study was to compare the effect of 6% hydroxyl ethyl starch solution with 4% gelatin and Ringer’s solutions on the haemodynamic stability of patients after coronary artery bypass graft (CABG) surgery and immediately after discontinuation of cardiopulmonary bypass (CPB).

**Methods:**

This was a randomised, double-blind clinical trial of 92 patients who were candidates for on-pump CABG. After discontinuation of CPB, all patients were transferred to the intensive care unit (ICU) and divided randomly into three groups. The first group received Ringer’s solution, the second group 4% gelatin, and the third 6% hydroxyl ethyl starch (HES) solution (Voluven). Haemodynamic parameters such as heart rate, mean arterial pressure, systolic blood pressure, diastolic blood pressure, central venous pressure, cardiac output and the presence of arrhythmias were documented.

**Results:**

The volume needed for maintaining normal blood pressure and central venous pressure in the range of 10–14 mmHg was less in the HES group than in the other groups. The volume was similar however in the gelatin and Ringer’s groups in the first 24 hours after surgery. Urinary output in the first four and 24 hours after surgery were significantly higher in the HES group than in the other two groups. Mean creatinine levels were significantly lower in the HES group.

**Conclusion:**

HES (6%) had a better volume-expanding effect than gelatin (4%) and Ringer’s solutions, and its short-term effects on renal function were also better than gelatin and Ringer’s solutions.

## Abstract

Immediately after coronary artery bypass graft (CABG) surgery, patients are haemodynamically unstable and need fluid support.^1^ The purpose of using volume expanders after cardiac bypass surgery is to maintain stable haemodynamics.^2^ Applying an appropriate fluid with enough volume at this stage may prevent systemic hypoperfusion and cellular hypoxia, which lead to systemic lactic acidosis.^3^ Furthermore, after cardiopulmonary bypass patients experience systemic inflammatory responses and endothelial damage, which lead to fluid extravasations and interstitial oedema. Therefore correct volume administration is recommended in this situation.^4^

There is controversy regarding the different types of solutions used after CABG, and various researchers have used materials such as crystalloid solutions or colloids, including albumin and gelatin, or other agents such as hydroxyl ethyl starch solutions. Volume expansion is an important aspect of these solutions, however, side effects, such as inflammatory responses, and effects on endothelial integrity and on organs such as the kidney should also be considered during their administration.^4^

Gelatins are polydispersed polypeptides produced by degradation of bovine collagen. Three types of modified gelatin products are now available: cross-linked or oxypolygelatins (e.g. Gelofundiol®), urea cross-linked (e.g. Haemacel®) and succinylated or modified fluid gelatins (e.g. Gelofusine®). Their molecular weight (MW) ranges from 5 000–50 000 Da, with an average of 30 000–35 000 Da. The various gelatin solutions have comparable volume-expanding powers and all are said to be safe with regard to coagulation and organ function (including kidney function).^2^

Hydroxyl ethyl starch (HES) is a widely used plasma substitute for correcting hypovolaemia in cardiac surgery patients. HES preparations vary with regard to concentration, mean MW, molar concentration, C_2_:C_2_ ratio, and solvent. HES solutions with a low MW and a low molar concentration are thought to be safe with regard to coagulation, and increased bleeding tendency no longer appears to be a problem (Valoven, HES 6%), even when higher doses are given.^3^

Some authors believe that albumin has a better volume-expanding effect than HES.^5^ Rehm *et al*. have shown that HES and albumin solutions caused mild systemic acidosis in patients undergoing normovolaemic haemodilution after cardiac surgery.^6^ Others maintain that a short time of infusion of a rapidly degradable HES solution after cardiac surgery produces impairment in fibrin formation and clot strength in thrombo-elastometry tracings. In this clinical setting, human albumin does not impair homeostasis.^7^

Correcting hypovolaemia with HES has been suggested to be associated with an increased risk of acute renal failure, and interest has recently been focused on the influence of HES solutions on renal function.^8^ Boldt *et al*. found better kidney function and less inflammation with the use of HES than with albumin solutions.^4^

The aim of this study was to compare the effect of 6% hydroxyl ethyl starch solution with 4% gelatin and Ringer’s solutions on haemodynamic stability of patients after CABG surgery and immediately after discontinuation of cardiopulmonary bypass.

## Methods

This was a prospective, randomised, double-blind clinical trial in 92 patients who were candidates for on-pump CABG. The age range of patients was from 40 to 75 years. Exclusion criteria were left ventricular ejection fraction < 40%, right heart failure, emergency patients, pump time > 180 minutes and clamp time > 90 minutes, patients who needed re-operation within the first six hours due to surgical haemorrhage or other reasons, renal failure needing haemodialysis, and those with respiratory failure.

All patients received pre-anesthesia medication. Lorazepam (1 mg orally) was given the night before the operation and intramuscular morphine (0.1 mg/kg) one hour before induction of anaesthesia in all patients. In the operating room, lidocaine (1%) was used for access to arterial and peripheral vessels and Ringer’s crystal solution was administered in a dose of 5–10 ml/kg. Anaesthesia induction was started with intravenous medazolam sufentanyl and pancranium.

After the use of 100% oxygen by mask, patients were intubated with an endotracheal tube and connected to a mechanical ventilator and central venous pressure (CVP) was introduced in the right internal jugular vein. Maintenance of anaesthesia was achieved with continuous infusion of idazolam, atrocurium and sufentanyl. After infusion of 300 IU/ kg heparin, the patient went on-pump and the activated clotting time (ACT) was above 480 s, mean arterial pressure 60–70 mmHg, haematocrit level was 22–27%, and the temperature was set at 32°C.

After discontinuation of cardiopulmonary bypass (CPB) all patients were transferred to the intensive care unit (ICU) and were randomly divided into three groups. The first group received Ringer’s solution, the second gelatin (4%), and the third group hydroxyl ethyl starch solution (HES) (6%) (Voluven) as a volume expander to maintain the CVP between 7 and 14 mmHg. Packed cells were infused where the haemoglobin level was lower than 8 mg/dl and fresh frozen plasma (FFP) was used for continuous bleeding with a normal range of ACT and APTT (activated partial thromboplastin time).

Cardiac output was monitored with a NICO instrument and haemodynamic values were monitored continuously. In situations where, after maintaining adequate volume, the mean arterial pressure was below 60 mmHg and cardiac index below 2 l/min/m^2^ body surface area, inotrope infusion (dobutamine or epinephrine) was started.

Haemodynamic parameters such as heart rate, mean arterial pressure, systolic blood pressure, diastolic blood pressure, central venous pressure, cardiac index and the presence of arrhythmias were documented. Other independent variables such as urinary output, serum electrolytes and serum creatinine levels were measured immediately after discontinuation of CPB, before transferring the patient to the ICU, immediately after arriving in ICU, and after two, four, six, 12 and 24 hours in ICU.

Study approval was obtained from the ethics committee of our Centre and written informed consent was obtained from the patients. The data were put into spreadsheets and comparison of variables between groups was done using Chi-squared or ANOVA tests.

## Results

Biometric data were similar in all groups. Mean anaesthesia time, pump time and cross-clamp time were the same in all three groups (Table 1). There were no mortalities in any of the groups. There were no significant differences in systolic and diastolic blood pressure between the three groups, and haemoglobin, blood urea nitrogen (BUN), creatinine, Na and K levels, partial thromboplastin time (PTT), and international normalised ratio (INR) were same in the three groups. No case was excluded from this survey and no significant differences were found between groups for mean arterial pressure, central venous pressure and heart rate (Table 2).

**Table 1.** Demographic Characteristics Of Patients (± SD)

	*Ringer’s solution (n = 29)*	*Gelatin (4%) (n = 31)*	*HES (6%) (n = 32)*	p-*value*
Age (year)	59 (11)	60 (8.7)	57 (10.4)	0.495
Weight (kg)	73.4 (10.8)	72.5 (11.9)	74.4 (11)	0.795
Height (cm)	167.4 (8.2)	165 (8.3)	167 (6.7)	0.750
Ejection fraction %	41 (8.4)	45 (6.7)	46 (5.9)	0.195
Numbers of bypass	3 (0.3)	3 (0.4)	2.9 (0.4)	0.449
Drug usage:				
Plavix	1	4	0	0.283
Beta-blocker	26	22	21	0.78
ASA	17	19	18	0.410
ACE inhibitors	15	16	17	0.380
Nitrates	14	12	13	0.210
Oral antidiabetic agents	7	8	7	0.150
Other antihypertensive agents	5	6	5	0.110
Diuretics	13	11	12	0.225
Anesthesia time (min)	263 (191–310)	250 (181–301)	247 (185–305)	0.140
CPB time (min)	109 (37)	99 (28)	106 (34)	0.120
Cross-clamp time (min)	63 (26)	55 (20)	59 (25)	0.170
Systolic BP (mmHg)	120 (11)	123 (16)	114 (9)	0.211
Diastolic BP (mmHg)	75 (11)	73 (10)	70 (9)	0.293
Na (meq)	134 (7)	140 (9)	135 (20)	0.212
K (meq)	4.3 (0.44)	4.3 (0.47)	4.35 (0.45)	0.143
PTT (s)	30 (4.5)	35 (5.3)	29 (5)	0.136
INR	1.1 (0.25)	1.08 (0.2)	1.08 (0.16)	0.278
Haemoglobin (g/dl)	12.7 (8.1–15)	12 (9–14)	12.3 (8.4–13.5)	0.323
BUN (g/dl)	13 (9–23)	15 (9–31)	15.5 (9–23)	0.275
Creatinine (g/dl)	0.9 (0.7–1.04)	0.95 (0.5–1.3)	1(0.7–1)	0.340

CBP: cardiopulmonary bypass, BP: blood pressure, ACE: angiotensin converting enzyme, PTT: partial thromboplastin time.

**Table 2.** Comparison Of Determined Variables Between The Three Groups (± SD)

	*Ringer’s solution*	*Gelatin (4%)*	*HES (6%)*	p-*value*
MAP after pump	61 (4)	63 (4)	64 (4)	0.410
MAP after moving to ICU	62 (3)	61 (3)	63 (4)	0.380
MAP after 2 hours in ICU	64 (4)	67 (3)	68 (4)	0.395
MAP after 4 hours in ICU	67 (5)	69 (6)	71 (7)	0.295
MAP after 6 hours in ICU	69 (5)	73 (4)	74 (7)	0.220
MAP after 12 hours in ICU	74 (9)	73 (11)	75 (10)	0.345
MAP after 24 hours in ICU	73 (7)	71 (4)	75 (5)	0.275
HR after moving to ICU	62 (3)	64 (5)	68 (6)	0.175
HR after 2 hours in ICU	73 (7)	74 (5)	72 (4)	0.195
HR after 4 hours in ICU	77 (7)	81 (6)	78 (5)	0.170
HR after 6 hours in ICU	75 (7)	80 (6)	78 (6)	0.220
HR after 12 hours in ICU	77 (7)	79 (5)	80 (6)	0.230
CVP after pump	11 (10–14)	12 (10–14)	12 (10–14)	0.270
CVP after moving to ICU	12 (10–14)	11 (10–14)	13 (10–14)	0.215
CVP after 2 hours in ICU	13 (10–14)	12 (10–14)	11 (10–14)	0.179

MAP: mean arterial pressure, HR: heart rate, CVP: central venous pressure

The volume needed for maintaining normal blood pressure and central venous pressure in the range of 7–14 mmHg was less in the HES group than in the other groups, but similar in the gelatin and Ringer’s groups in the first 24 hours after surgery. Urinary output in the first four and 24 hours after surgery was significantly higher in the HES group than in the other two groups.

Mean creatinine levels on the first day post operation were 1.25 ± 0.23 mg/dl in the Ringer’s group, 1.3 ± 0.24 mg/dl in gelatin group and 1.06 ± 0.13 mg/dl in the HES group. On the second day post operation, these values were 1.4 ± 0.25, 1.41 ± 0.26, 1.13 ± 0.16 mg/dl in the Ringer, gelatin and HES group, respectively. Mean creatinine levels were significantly lower in the HES group (Table 3).

**Table 3.** Comparison Of Determined Variables Between The Three Groups (± SD)

	*Ringer’s solution*	*Gelatin (4%)*	*HES (6%)*	p-*value*
Mean volume infused during surgery (ml)	2150 (340)	1925 (290)	1320 (250)	0.011
Mean volume infused in first 24 hours in ICU (ml)	6100 (400)	5300 (380)	3500 (210)	0.001
Units of packed cells infused in 24 hours in ICU	94	93	96	0.275
Units of FFP infused in 24 hours in ICU	53	53	48	0.170
Units of platelets infused in 24 hours in ICU	28	34	23	0.145
Amount of haemorrhage in first 24 hours (ml)	1300 (260)	1350 (270)	1280 (280)	0.170
Amount of urine output in first 4 hours in ICU (ml)	1700 (180)	1760 (190)	2250 (290)	(0.02)
Amount of urine output in first 24 hours in ICU (ml)	4450 (310)	4520 (340)	5200 (330)	(0.03)
Creatinine in first postoperative day (mg/dl)	1.32 (0.23)	1.31 (0.24)	1.06 (0.13)	0.004
Creatinine in second postoperative day (mg/dl)	1.4 (0.25)	1.41 (0.26)	1.13 (0.16)	0.004

There were no significant differences between the three groups in the amount of blood, FFP and platelet transfusions in the ICU (Table 3). Arrhythmias in ICU, extubation time and ICU stay were the same in all groups (Table 4).

**Table 4.** Comparison Of Determined Variables Between The Three Studied Groups (± SD)

	*Ringer’s solution*	*Gelatin (4%)*	*HES (6%)*	p-*value*
Extubation time (min)	452 (418–508)	445 (410–500)	463 (420–430)	0.215
ICU stay time (hours)	46 (42–48)	47 (43–48)	45 (42–48)	0.175
Arrhythmias in ICU (n)	2	0	2	0.459

## Discussion

The main result of this study was that haemodynamic stability could be achieved after CABG surgery with less volume of HES than gelatin and Ringer’s solutions. The kidney function was better in the short term in the HES group than in the other two groups.

In our centre, we selected adult patients for CABG. We are aware that enough of a suitable volume expander is needed for haemodynamic stability after CABG, and that some volume expanders have side effects. Patients usually have a systemic inflammatory response after CPB for CABG and the resultant endothelial damage leads to hyperpermeability and interstitial oedema.^4^

Some researchers have shown that HES reduced inflammation and endothelial damage.^4^ It also maintained the cell’s integrity and function.^4^,^9^ Lower-molecular weight HES molecules had an effect on the arteriolar integrity and could reduce arteriole-induced oedema in clinical and experimental models.^10^ Reported effects of HES usage were improvement in the microcirculation and the oxygenation of organs.^11^ HES also had an effect on inflammation. Reduction of macrophage inflammatory protein (MIP-2), IL-1β and TNF-α levels was found to be a mechanism for reduction of inflammation after the use of HES.^12^

The most dangerous complication after CABG is kidney damage and some researchers demonstrated kidney damage after the use of HES but found gelatin (4%) to be safer.^8^ Others have shown little reduction in glomerular filtration rate (GFR) after the use of high-molecular weight HES.^13^ Boldt *et al*. reported a lower inflammation rate and better GFR with HES.^4^

In our study there was better haemodynamic stability with lower volumes of HES. Renal function was good after its use in the first two days after CABG, which indicates that renal function can be maintained after use of 6% HES. Other reporters have shown less renal damage after the use of HES than with gelatin, albumin and Ringer’s solutions.^4^

In our study we used less volumes of HES than Ringer’s solution and gelatin and this produced a better volume-expanding effect with HES than with gelatin and Ringer’s solutions. Better oxygenation and lower serum lactate concentration were shown after the use of HES than with gelatin.^15^ There were no differences between the three groups as far as mortality rate is concerned.

There were some limitations to the study. Because of the systemic inflammatory response after CPB, it would have been advisable to compare inflammatory biomarkers in the three groups but this was not done. It has been reported that HES had an effect on the acid–base balance in some studies, but this was not determined in our study.

## Conclusion

Our study showed that HES (6%) had a better volume-expanding effect than gelatin (4%) and Ringer’s solution, and its short-term effects on renal function were also better than with gelatin and Ringer’s solution.
